# Cancer

**Published:** 1916-08

**Authors:** 


### Cancer.

In response to numerous propagandist statements, we wish to say, with equal dogmatism though not of a personal kind, that cancer is NOT a local condition, primarily. True, like every other disease, it must have a local habitat and this is almost always limited in extent for a considerable period but the disease is no more essentially local than diphtheria or gout. This is not a quibble of words. Every hope as to prompt diagnosis, prophylaxis of the disease itself and of therapeutics beyond sacrificial removal or destruction of the part involved, depends upon the conception of cancer as a general disease.

In a purely academic sense and as representative of ideal conditions, toward the realization of which very little progress has been made, early cancer may be a curable condition —in the sacrificial sense stated. Under existing conditions, cancer is not curable even in this sense excepting in a comparatively small minority of cases affording exceptionally favorable opportunities for early recognition. Nor are the great majority of cases which are not subjected to early surgical or other destructive methods of treatment, delayed through lack of skill or neglect by diagnosticians, according to standards at present possible.

Speaking approximately, the best surgical statistics of exceptionally favorable series show that about 25% of cases attacked by the operator with the hope of radical success are cured. Surgical statistics of series attacked as potentially curable, actually give about 10% of cures. There are no statistics, so far as we know, to indicate what percentage of cancer cases in the aggregate or what percentage of cases excluding those in which diagnostic methods are neglected or advice as to operation disregarded, are ultimately cured. The guess may be hazarded that 1% of all cases and 2% of cases in which no gross neglect can be ascribed to patient or physician or operator, are cured.

Such statistics are not made worse by rarity of material which excuses unfamiliarity with potential diagnostic and therapeutic problems and lack of attainable skill in either of these directions. Neither, if we exclude instances of gross diagnostic and operative incompetence, is there a marked difference in results, comparing average medical and surgical practitioners, with authorities. Cancer deaths are about half as frequent as those from tuberculosis, nearly seven tenths as frequent, if we exclude deaths under the age of five, for which period cancer is very rare. Nearly 1% of all deaths are from skin cancers, where the difficulties of diagnosis and treatment would seem to be at a minimum.

The great majority of cancers develop at an age and under other circumstances which suggest the diagnosis even to the laity. Instead of there being danger of forgetting that cancer may be present, the physician is rather embarrassed at having the diagnosis suggested almost as a routine and in the most varied conditions, including those of trivial nature. From the literary standpoint, the profession, not to mention the laity, has had fully as much education as regards cancer as for tuberculosis. Tuberculosis has been prevented or cured to an extent corresponding to the reduction of the general death rate. That is to say, while the general death rate has been reduced from 20 to 14, the tuberculosis fraction remains just about 10% of the total. Cancer, on the other hand, seems to be increasing, although this may be due to the greater average longevity of the race. While there has been a decided improvement in statistics of favorable operative series, this improvement seems to be less than the corresponding improvement in the results of treatment of tuberculosis. The complaint of surgeons—and propagandists—that cancer cases are not referred sufficiently early for radical treatment, is as bitter as it was 20 years ago and, still more significant, comparative results of series according to lapse of time from the beginning of symptoms—excluding obviously neglected cases—do not show the significant superiority of the chances for cure after very early operation, as contrasted with progressively longer periods, within reasonable limits. (Bloodgood).

The logical conclusion would seem to be that, on the whole, the profession, diagnostically and therapeutically, is doing the best it can under existing limitations of scientific knowledge and that, under these conditions, cancer is not a preventable disease except to a very minor degree and in particular groups of cases, while it is not curable except in a small percentage of cases, and then by sacrifice of the area attacked. It is desirable that the profession should recognize its limitations and look ahead to future accomplishments. But we feel strongly that the time is not ripe for optimistic declarations of what ought to be done; certainly not for stating these in terms of what can be done. It is most unfortunate that optimism should be so expressed and so published as to reflect upon the skill and caution of the profession.

